# Supplementary material for: Effect of bariatric and metabolic surgery on rheumatoid arthritis outcomes: A systematic review
Source: PLoS One. 2023 Nov 17;18(11):e0294277. doi: 10.1371/journal.pone.0294277 (PMC10655969; doi:10.1371/journal.pone.0294277)
Supplement: S1 Table — (DOCX) [file pone.0294277.s002.docx]

| **Supplementary table. Main characteristics and results of the selected studies aiming to evaluate the outcomes of bariatric surgery on RA patients** | | | | | |
| --- | --- | --- | --- | --- | --- |
| **First author (ref)**  **Yr of publication**  **Country** | **Study design**  **Population: cases (mean age, yrs); controls (mean age, yrs)** | **Inclusion criteria/ exclusion criteria** | **Methods** | **Main outcomes** | **Conclusion**  **of the authors** |
| **Sparks et al**  **2015**  **Boston** | Cohort study  Cases (surgery)  53 RA (47.9 (10.5) years);  No control group | *Inclusion criteria*  .Diagnosis of RA according to the 1987 American College of Rheumatology  .RA patients who underwent bariatric surgery (Roux-en-Y gastric bypass, sleeve gastrectomy, laparoscopic adjustable gastric banding, or other absorption limiting gastric procedures)  .Adequate clinical data available three months prior to surgery and after surgery | *Collected data*  *.* RA characteristics (serologic status, bone erosions on radiography, and disease duration)  . Disease activity measures : symptoms, tender/swollen joint count, laboratory data, and global clinical assessment, DAS28VS, DAS28CRP,CDAI,SDAI, RAPID  . RA-related medications  .Anthropometric data (BMI)  *Follow-up*  Index date (bariatic surgery), baseline (prior to bariatric surgery), 6M (±2), 12 M (±2) post-bariatric surgery and most recent follow-up.  *Endpoint*  .Changes in anthropometrics, laboratory values, RA disease activity, and RA-related medication use between each post-surgical time point and baseline values | .Substantial weight loss (mean 41.0 kg [SD 17.3]) at 12 months post-surgery *P*<0.001 compared to baseline.  .Significantly lower erythrocyte sedimentation rate, C-reactive protein, and RA-related medication use at follow-up visits compared to baseline (*P*<0.05).  .RA disease activity category measures were significantly lower at all follow-up time points compared to baseline (*P*<0.001)  .Twelve months after bariatric surgery, 68% of subjects were in remission compared to 26% at baseline (*P*<0.001). | .Weight loss may be an important non-pharmacologic strategy to reduce RA disease activity. |
| **Fang et al**  **2020**  **China** | Cohort  65 patients  Cases (Surgery)  32 (51.8(11.8) years);  Control group  33 (57.6 (16.4) years) | *Inclusion criteria*  .RA diagnosis determined by 2010 ACR/EULAR classification criteria  .Age of 18–65 years  .BMI ≥30 kg/m²  .No abuse of alcohol or psychotropic drugs, and no serious behaviour disorder  or mental retardation  .TJCs ≥5 and SJCs ≥3  .ESR ≥20 mm/h  .CRP ≥20 mg/L.    *Exclusion criteria*  .History or current infection or any type of malignant cancer  Secondary obesity  (cushing syndrome, hypothyroidism, polycystic ovary syndrome, hypopituitarism, hypothalamic obesity,  or prolactinoma)  .Intolerance to surgery due to serious organ diseases. | *Collected data*  *.* RA characteristics  . Disease activity measures : symptoms, tender/swollen joint count, laboratory data, and global clinical assessment, DAS28VS, DAS28CRP,CDAI, RA-related medications  .Anthropometric data (BMI)  *Follow-up*  .Baseline,4,8,12 months post surgery  *Endpoints*  ACR 20 , 50, 70 responses, Disease activity DAS28 VS, DAS28 CRP, CDAI, medication tapering at 12 months  However, medication use did not differ between the 2 groups at baseline or at the 12-month  follow-up (all p > 0.05). Medication tapering for RA patients who underwent bariatric surgery was,  therefore, not superior to that in non-surgical patients | .ACR20, ACR50 and ACR70 were 75.0% vs. 51.5%, 53.1% vs. 39.4% and 31.3% vs. 21.2% in the bariatric surgery and non-surgery groups, respectively (all p < 0.05).  . Significant decrease in the  DAS28-ESR, DAS28-  CRP and cDAI compared to baseline (all p < 0.05).  . A significant reduction was observed in the use of leflunomide, biological agents,  combination treatments, and NSAIDs in both groups (p < 0.05 or p < 0.01)  . No difference in medication use between the 2 groups (all  p > 0.05).  There were no statistically significant differences among the 2 groups for any of the aforementioned variables (p > 0.05) except for PtGA (p = 0.03). | Weight loss after bariatric  surgery was associated with lower disease activity.  Medication tapering for RA in patients who  underwent bariatric surgery was not superior to that in non-surgical patients. |
| **Lin et al**  **2022**  **Taiwan** | Retrospective  33,075 patients  Cases (surgery) 6615 (53.2 (0.14) years);  Control group 26,460 (54.3(0.07) years) | *Inclusion criteria*  *. RA according to the* International Classification of Diseases,  .Age ≥ 18 years old  .Morbid obesity  *Exclusion criteria*  .Age were < 18  .Missing significant covariates | *Collected data*  *.*Sociodemographic, major comorbidities  *Endpoint*  .Incidence of major morbidities, in-hospital mortality | .Prior bariatric surgery was significantly and independently  associated with reduced odds for any morbidity and in-hospital mortality as compared  with no prior bariatric surgery 36.5% vs 54.6%, 0.45, 95% CI (0.42, 0.48), p< 0.001) and (0.4% vs 0.9%, 0.41, 95% CI (0.27–0.61), p < 0.001) respectively.  .Significantly lower odds for  having unfavorable discharge and prolonged length of stay (LOS) as compared with those without bariatric surgery (aOR:  0.43, 95% CI: 0.39–0.46, p < 0.01) | Prior bariatric surgery is associated with better in-patient outcomes among RA, including in-hospital mortality,  major morbidities, discharge destination, and LOS. |

*RA: rheumatoid arthritis, csDMARDs: conventional disease-modifying anti-rheumatic drug , NSAIDs: non-steroidal anti-inflammatory drugs, M:months, DAS28: 28-joint count disease activity score; ESR: erythrocyte sedimentation rate, CRP: C-reactive protein, cDAI: clinical disease activity index, PtGA: patient’s global assessment of*

*overall well-being;LOS: length of stay;*
