# Supplementary material for: Effect of bariatric and metabolic surgery on rheumatoid arthritis outcomes: A systematic review
Source: PLoS One. 2023 Nov 17;18(11):e0294277. doi: 10.1371/journal.pone.0294277 (PMC10655969; doi:10.1371/journal.pone.0294277)
Supplement: S2 Table — (DOCX) [file pone.0294277.s003.docx]

**Table 1:** Risk of bias assessment according to the Newcastle Ottawa Scale (NOS) for the cohort studies

|  | **Selection** | **Comparability** | **Outcome** |
| --- | --- | --- | --- |
| Fang Xu et al, 2020 | *** | * | *** |
| Jefferey et al, 2015 | *** | * | ** |
| Chen lin et al, 2022 | **** | * | *** |
